# Resource Elasticity of Offspring Survival and the Optimal Evolution of Sex Ratios

**DOI:** 10.1371/journal.pone.0053904

**Published:** 2013-01-23

**Authors:** Rui-Wu Wang, Ya-Qiang Wang, Jun-Zhou He, Yao-Tang Li

**Affiliations:** 1 Kunming Institute of Zoology, Chinese Academy of Science, Kunming, Yunnan, China; 2 School of Mathematics and Statistics, Yunnan University, Kunming, Yunnan, China; 3 Statistics and Mathematics College, Yunnan University of Finance and Economics, Kunming, Yunnan, China; Institut Pluridisciplinaire Hubert Curien, France

## Abstract

The fitness of any organisms includes the survival and reproductive rate of adults and the survival of their offspring. Environmental selection pressures might not affect these two aspects of an organism equally. Assuming that an organism first allocates its limited resources to maintain its survival under environmental selection pressure, our model, based on the evolutionarily stable strategy theory, surprisingly shows that the sex ratio is greatly affected by the environmental pressure intensity and by the reproductive resource elasticity of offspring survival. Moreover, the concept of the resource elasticity of offspring survival intrinsically integrates the ecological concepts of *K* selection and *r* selection. The model shows that in a species with reproductive strategy *K*, increased environmental selection pressure will reduce resource allocation to the male function. By contrast, in a species with reproductive strategy *r*, harsher environmental selection pressure will increase allocation to the male function. The elasticity of offspring survival might vary not only across species, but also across many other factors affecting the same species (e.g., age structure, spatial heterogeneity), which explains sex ratio differences across species or age structures and spatial heterogeneity in the same species.

## Introduction

The classical definition of fitness deals separately with the survival and reproductive functions of the organism, and with males and females, which partly avoids the problem of resource allocation. The fitness of an organism is simply described as 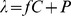, where 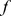 is the specific reproductive rate (reproductive rate represents the number of offspring produced by an organism), 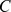 is the survival from birth (or the seedling stage) to the adult or reproductive stage, and 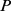 is the survival (per time interval) of the reproductive stage [1]. In this original definition of organism fitness, the resources of the organism are linearly correlated to survival and reproduction, as well as to the male and female functions [1]–[3]. On this assumption, the survival (or reproduction) and the sex ratio are independent of the resource allocation [4], and also independent of mortality, fecundity with age, size, and environmental selection pressures [5].

However, the fitness of any organism, comprising the survival and reproductive rate of adults and the survival of their offspring, should depend on the allocation of limiting resources [6]. If the available resources are limited, the increase in one activity must be at the expense of another, constituting a trade-off in resource allocation. In this context, the trade-off in resource allocation will exist between survival and reproductive effort, and between the male and female functions. This dilemma of resource allocation has received considerable attention and each aspect has spawned a huge literature [6]–[8]. The consensus opinion is still that survival and reproduction should be addressed separately, as if they were independent questions, and only reproductive effort and sex allocation have been incorporated into a single framework [8], [9]. However, an interaction between these two forces of natural selection must exist because male function, female function and survival are undoubtedly constrained by a single resource pool. Under natural conditions, if there exists a discriminative selection pressure against survival, male and female function, such as inter- or intra-specific competition or predation/parasitism, the increased resource allocation to one of these three functions must reduce the resources invested in the other one or two functions, and might therefore affect sex allocation in a nonlinear process [8].

The variation of the resource allocation to either survival or reproduction, however, might also lead to the survival or reproduction rate change. This is the resource elasticity of survival or reproduction (elasticity here could be defined as percentage change in survival or reproduction in response to a one percent change in resource allocation), similar to elasticity concept in economics (see Appendix S1) [10]. Unfortunately, there exist few literatures dealing with whether or not such resource elasticity of survival or reproduction could affect sex ratio of any organisms. This being the case, it is necessary to develop a model of the comprehensive fitness characteristics of an organism that incorporates the interactions between sex allocation and resource elasticity in a single framework.

In this model, we assumed that the resource available to an organism is limited. Considering the resource elasticity of survival, we explored the optimal resource allocation to survival and reproduction and to producing male and female offspring. We asked the following question: under an environmental selective pressure, such as competition or predation, should the individuals/species of different reproduction strategies take different resource allocation strategies to maximize their total fitness gain?

## Methods

### Model of Discriminative Environmental Pressure on Fitness Functions

Throughout this article, we use population growth rate (it could also be defined as reproductive factor) as the measure of the fitness [11]–[14], and the key assumption is that some underlying resource is limited, such as energy or any other resource that can be spent only once. In addition, an individual was assumed to reach its reproductive maturity after a single time interval (i.e., from *T* to *T+1*). In a stochastic environment, it is possible that the organisms will first allocate their limited resource to their survival under environmental pressure, and then the remainder to their future reproduction. Therefore, in this model, we assume that the resource allocation to survival will increase under harsher environmental pressure, whereas the resource allocation to reproduction will monotonically decrease to zero because of the limited availability of resource. Therefore, the resource allocation is nonlinearly related to reproduction. In this comprehensive model, the functions of survival, male reproduction, and female reproduction will be constrained by the trade-off in resource allocation. We would like to find the optimal sex allocation between the male and female functions of an organism subject to the optimization of fitness gain under environmental selection pressure.

### Optimal Resource Allocation under Discriminative Environmental Selection Pressure

As a previous model has demonstrated that density-dependent effect has no impact on the sex ratio in a resource allocation model [15], in this article we suppose that individual organisms have no density-dependent effect on offspring production, and that offspring production also has no density-dependent effect on the survival of the adult individuals. All of the aspects of the fitness of an organism are mainly determined by resource allocation. The total resource of each individual is affected by environmental selection pressure, and we assume the available resource is first allocated to individual survival under the environmental selective pressure, which means that the reproduction allocation will decrease as the environmental selective pressure increases. In the total resource of each individual allocated to the three competing functions of male production, female production, and survival, let each individual allocate a proportion 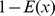 to survival, and the remaining proportion 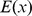 to reproduction. Among the proportion of resource 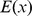, a proportion 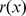 is allocated to male and 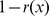 to female, where 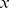 denotes the strength of environmental pressure. Then the number of organisms at time *T+1* can be written as:
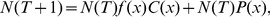
(1)where *N*(*T*) is the number of organisms at time *T*, 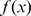 is the number of female offspring or plant seeds produced by an organism, *C*(*x*) is the probability of an offspring survival from *T* to *T+1*, and *P*(*x*) is the probability of an adult survival from *T* to *T+1*. The notations used in this and later sections are listed in Table 1.

**Table 1:** Definitions of the notations used throughout this paper.

Symbol	Definition
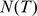	The number of organism individuals at time *T*
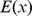	The proportion of total resource allocated to reproductive effort for an individual organism
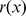	The proportion of total reproductive resource allocated to male reproductive effort for an individual organism
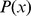	The probability of adult survival from *T* to *T+1*
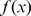	The number of females produced by an individual organism
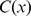	The probability of an offspring survival from *T* to *T+1*

From (1), the fitness of an organism can be written as
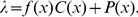
(2)For mathematical simplicity, we assume that female reproduction is a linear function of its resource input, that is, the female reproduction 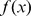 can be written as:
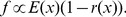
(3)Let
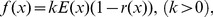
(4)where 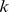 represents the maximum number of female offspring produced by an organism individual when all available resources allocated for reproduction are spent on female production. Here the probability of an offspring survival 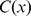 could be a function of any other parameters. For example, if 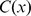 is a function of the reproductive allocation *E*(*x*), it can be denoted by 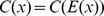.

If the available resource is first allocated to individual survival under the environmental selective pressure, then the effect of the environmental selective pressure on individual survival can be neglected and the resource allocation to reproduction will monotonically decrease to zero, so we can suppose
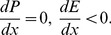
(5)


From equations (2) and (4), we know that the population growth rate 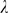 depends on the resource allocation strategy 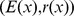. If we assume that the resource allocation strategy 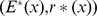 is an ESS, we must have 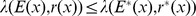 for any allocation strategies 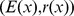. If the 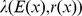 is donated by 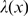 and 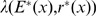 is donated by 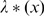, then, 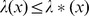.When the allocation pattern 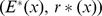 is an optimal strategy, the population growth rate 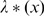 attains its maximum. From optimal theory, a necessary condition for the population growth rate 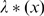 to attain the maximum is 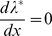 and to ensure the population growth rate 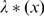 is a maximum rather than a minimum, the condition 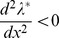 is therefore required.

Differentiating equation (2) with respect to *x*, we get
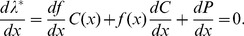
(6)Substituting equations (4) and (5) into equation (6), we can derive the following equation:
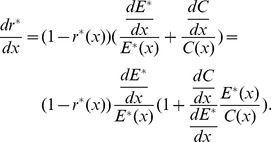
Let 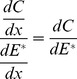,

Therefore, we have
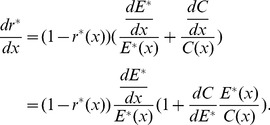
(7)From (7), it is easy to determine that the sign of 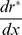 is decided by (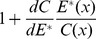) because 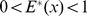, 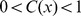, 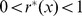, and 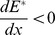. Therefore, the characteristics of 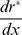 will be predominantly determined by 
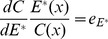
, where 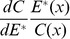 is the percentage change in offspring survival divided by the percentage change in resource allocation. This can be defined as the resource elasticity of offspring survival. This is very similar to the elasticity concepts of economics, such as the well-known price elasticity of demand [10]. The resource elasticity of offspring survival is a measure used to show the responsiveness of offspring survival to a change in resource allocation, more precisely, it gives the percentage change in offspring survival in response to a one percent change in resource allocation.

## Results

**Figure 1:**
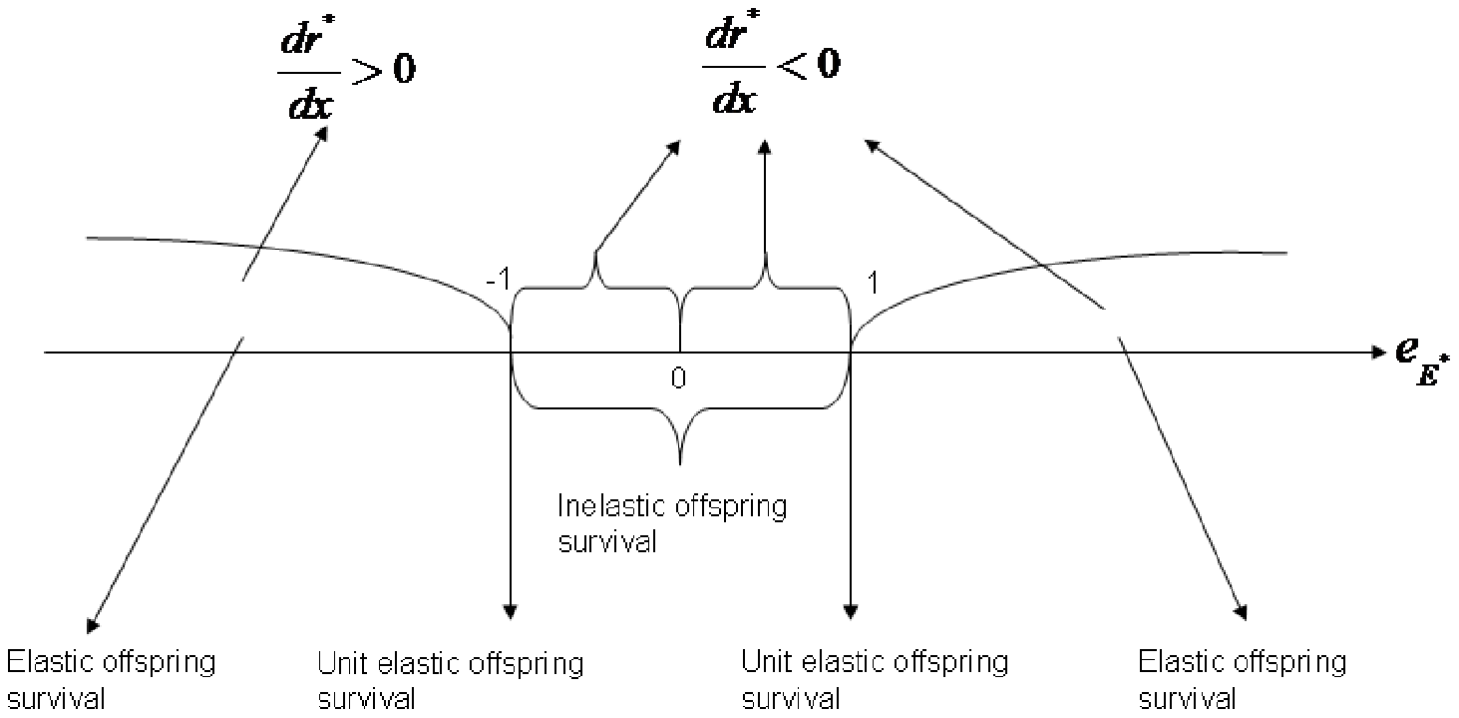
The effect of the resource elasticity of the offspring survival parameter 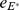 on the sex ratio (male) under environmental selection pressure. When 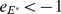, the increased environmental selection pressure will increase the proportion of males, namely, 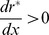. When 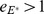, the increased environmental selection pressure will reduce the proportion of males, namely, 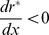. When 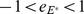, the increased environmental selection pressure will reduce the proportion of males, namely, 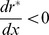.

If 
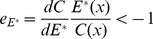
, from (7), we have 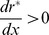, so that the resource allocation to the male function will increase with increasing environmental selection pressure. The model experimental result shows here that if 
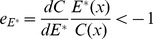
 (resource elasticity of offspring survival), the organism will allocate more resources to the male function to maximize its total fitness gain under increased environmental selection (Figure 1).

**Figure 2:**
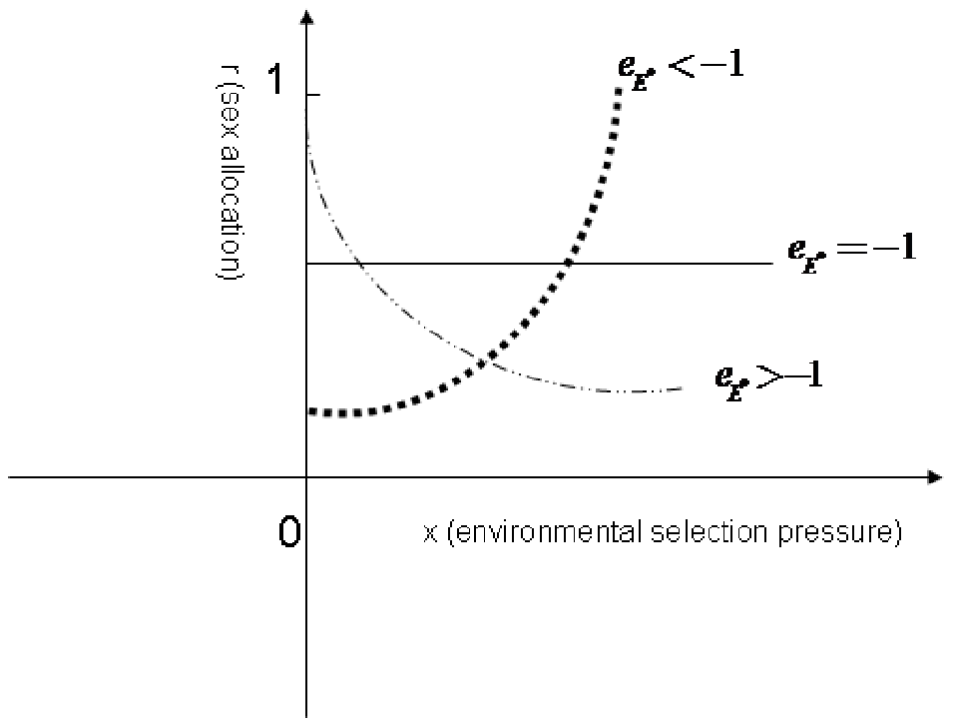
The effect of environmental selection pressure 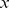 on the sex ratio 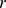 (male proportion) with different values for the resource elasticity of offspring survival.

If 
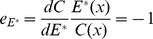
, the sex ratio will be constant under the influence of environmental factors (Figure 2).

Similar to the price elasticity of demand (see Appendix S1), If 
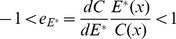
, the offspring survival is not very responsive to the change of resource allocation to reproduction. The offspring survival is inelastic to resource allocation for reproduction in such condition. From (7), we have 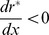, namely, the male function will decrease with increasing environmental selection pressure. The model experiment result shows here that if 
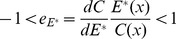
 (inelastic survival of offspring), the organism will allocate less resource to the male offspring under such condition.

If 
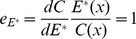
, which is similar to unitary elasticity in economics, from (7), we also have 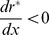, so resource allocation to the male function will decrease with increasing environmental selection pressure *x*.

If 
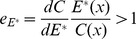
, from (7), we have 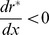, so the resource allocation to the male function will decrease with increasing environmental selection pressure *x*. The model experiment result shows here that if 
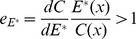
 (resource elasticity of offspring survival), the organism will allocate less resource to the male function to maximize its total fitness gain under the increased environmental selection.

## Discussion

Sex ratio evolution is closely related to resource allocation, and some studies in the literature have dealt with how resource allocation affects sex allocation under different male and female function relationships [4], [9], [16]. However, the integration of environmental selection pressure and sex allocation has not been directly addressed, although it is partly implied in the models of Zhang et al. [4], [9]. The model described in this paper shows that if we assume that the environmental selection pressure will force an organism first to allocate its limited resource to its survival [1], [17], the evolution of the sex ratio will depend strongly on the environmental selection pressure, as well as on the resource elasticity of offspring survival. However, whether the resource elasticity of offspring survival affects the evolution of the sex ratio has never been addressed before.

Our model shows that the environmental selection pressure leading to the variation in the offspring sex ratio of the target organism is greatly affected by the resource elasticity of offspring survival, whereas the resource elasticity of offspring survival might be determined by the reproductive strategy of the organism. Constrained by the same resource pool, organisms with different genetic characteristics, evolutionary pathways, habitats, age structures, or any other related factors, might have different resource elasticity of offspring survival. Therefore, there may be different sex ratio investment rewards across different species or even across different genotypes, phenotypes, or even age structures within the same species [17], if male production is nonlinearly related to female production when constrained by limited resource availability. The difference in reproductive strategies is essentially that different reproduction strategies have different offspring survival probabilities with a given resource allocation [1], [18], [19]. The resource elasticity of offspring survival developed here effectively integrated the differences in offspring survival probability.

For organisms that quantitatively produce more offspring with increasing resource allocation to their offspring, the increase in the proportion of resource allocated to reproduction might reduce the percentage of offspring survival. This is *r* selection, in which the organism produces more offspring but offers less parental care or allocates less resource to offspring development, and therefore has a lower offspring survival probability [1], [19], [20]. In this model, that the allocation of more resource to reproduction reduces the offspring survival probability falls within the range of resource elasticity of offspring survival (i.e., left of the −1 point in Figure 1).

Contrary to *r*-selection organisms, the *K*-selection organisms, which allocate more of their resources to increasing their offspring survival [2], [18], [19], will fall within right of the resource elasticity of offspring survival in Figure 1 of this model. For *K* selection organisms, the parents offer more protection/care to their offspring, produce offspring with larger body sizes, or have longer gestation periods to allow the full development of their offspring, and so on. In this way, the *K*-selection organism might increase its survival but produce fewer offspring. Therefore, the resource elasticity of offspring survival for *K*-selection organisms might be greater than -1 (right of Figure 1), and the increasing environmental selection pressure will reduce the proportion of male offspring.

When the percentage of offspring survival is not greatly affected by changes in the percentage of resource allocation to reproduction, offspring survival will be inelastic to resource allocation for reproduction (i.e. 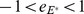), similar to the inelastic demand to price in economics [10]. In such situations, the sex ratio (proportion of males) will decrease slightly with increasing environmental pressure (Figure 2). When 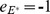, the sex ratio will be constant under the influence of environmental factors.

The sex ratio determination mechanism suggested here can help explain why the sex ratio varies contrarily across species under the harsher living environment. For example, the spotted eagle, which usually produced one or two offspring per year with *K* selection strategy, the male offspring proportion has increased in good year with rich food condition [20]. This is also true for primates which produce one or two offspring every one or two years. The birth sex ratios of primates are male biased in zoos in which the food availability and living condition are better than in wild condition [21]. However, the experimental data and empirical observations showed that house mice, a typical *r* selection species, will produce more male offspring than females when the mothers were underfed [21], [22]. The concept that sex ratio depends on environmental selection pressure as well as the resource elasticity of offspring survival well explains such contradictory sex ratio variation under the harsher food availability.

Although the resource elasticity or inelasticity of offspring survival was first introduced to explain biological characteristics, the concept of price/resource availability elasticity or inelasticity of demand, consumption, or any other behavior, is one of the most basic economic rules, and has been shown to greatly affect the behaviors of human beings [10]. Because the resource elasticity of offspring survival to reproduction resource might vary across different species because of differences in their reproductive strategies, the sex ratios of different species might be very different under the same environmental section pressure. Theoretically, the resource elasticity of offspring survival might also be a function of other physiological factors, and different environments might lead to different physiological responses. Therefore, this might result in different resource elasticity of offspring survival in the same species, which might in turn lead to the different sex ratios for the same species under different environmental selection pressures.

In terms of the biological characteristics of the sex ratio (allocation), the reproductive resource elasticity (or inelasticity) of offspring survival might be predominantly determined by the reproductive reaction to resource allocation, and this should be easier to understand. However, it is also theoretically possible that the resource elasticity (or inelasticity) of offspring survival is a reaction to changes in other related factors, such as hormone or chemical responses (e.g., nervous strain resulting from intraspecific or interspecific competition in animal, and both negative and positive allelopathic effects in plants). The resource elasticity (or inelasticity) of offspring survival might even be a response to age structure. In different age structures, the resource offspring survival might differ with a change in resource allocation because of variations in parent care or nutrient utilization [18]. Under the same environmental selection pressure, the resource elasticity (or inelasticity) coefficient of offspring survival might differ with different age structures, and therefore might also lead to a change in the sex ratio of a specific population. If the resource elasticity (or inelasticity) of offspring survival responds to different factors, the sex ratio (allocation) might change very differently or even in the opposite direction under changes in different components of the environmental selection pressure.

Throughout this paper, we assumed that available resources are limited and that there is a trade-off between survival and reproduction for resource allocation. Under such an assumption, we suppose that an organism allocates limited resources first to survival and then to reproduction. However, this will not always be the case for a species. When available resources are not limited for an organism, such as when a pioneer species with less species competition does not experience resource trade-off between survival and reproduction under increased environmental selection pressure, the sex ratio might not vary under different environmental pressure [4]. It is also true that the sex ratio pattern might be very different from the pattern predicted by this model if an organism does not first allocate limited resources to survival under increased environmental selection pressure. If an organism first allocates limited resources under environmental selection or extra availability of resources to reproduction and then to survival, the sex ratio variation will change to the pattern predicted by Trivers-Willard Hypothesis and the model of Zhang & Wang [4], [6]. Using sex ratio variation patterns for a species, the resource allocation strategy of the species can therefore be hypothesized.

## Supporting Information

Appendix S1
**Supporting information for “The concept of Elasticity”.** Elasticity is one of the most basic concepts in economics. Here, let's use the price elasticity of demand to illustrate this concept used in economics [10]. Price elasticity of demand is a measure used to show the responsiveness, or elasticity, of the quantity demanded of a good or service to a change in its price. More precisely, it gives the percentage change in quantity demanded in response to a one percent change in price (holding constant all the other determinants of demand, such as income. It can be described as: 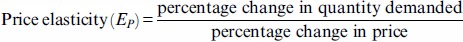. Since price and quantity demanded always move in opposite directions, 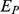 is a negative value. For convenience, however, the absolute value of 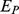 is used. If 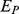 is larger than 1, we say demand is elastic: Consumer response is large relative to the change in price. If 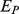 is less than 1, we say demand is inelastic: Consumers are not very responsive to price changes. If 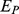 is equal to 1, demand is unitary elastic. In this case, the percentage change in quantity demanded is exactly equal to the percentage change in price.(DOC)
