# Supplementary material for: Resource Elasticity of Offspring Survival and the Optimal Evolution of Sex Ratios
Source: PLoS One. 2013 Jan 23;8(1):e53904. doi: 10.1371/journal.pone.0053904 (PMC3553167; doi:10.1371/journal.pone.0053904)
Supplement: Appendix S1 — Supporting information for “The concept of Elasticity”. Elasticity is one of the most basic concepts in economics. Here, let's use the price elasticity of demand to illustrate this concept used in economics [10]. Price elasticity of demand is a measure used to show the responsiveness, or elasticity, of the quantity demanded of a good or service to a change in its price. More precisely, it gives the percentage change in quantity demanded in response to a one percent change in price (holding constant all the other determinants of demand, such as income. It can be described as: . Since price and quantity demanded always move in opposite directions, is a negative value. For convenience, however, the absolute value of is used. If is larger than 1, we say demand is elastic: Consumer response is large relative to the change in price. If is less than 1, we say demand is inelastic: Consumers are not very responsive to price changes. If is equal to 1, demand is unitary elastic. In this case, the percentage change in quantity demanded is exactly equal to the percentage change in price. (DOC) [file pone.0053904.s001.doc]

**Appendix S1** Supporting information for “The concept of Elasticity”

Elasticity is one of the most basic concepts in economics. Here, let’s use the price elasticity of demand to illustrate this concept used in economics [10]. Price elasticity of demand is a measure used to show the responsiveness, or elasticity, of the quantity demanded of a good or service to a change in its price. More precisely, it gives the percentage change in quantity demanded in response to a one percent change in price (holding constant all the other determinants of demand, such as income. It can be described as:

.lercentage change ine elasticity of demand refers to the percentage change in quantity demanded divided by the percentage changPrice elasticity ($E_{P}$)= $\frac{\text{percentage change in quantity demanded}}{\text{percentage change in price}}$.

Since price and quantity demanded always move in opposite directions, $E_{P}$ is a negative value. For convenience, however, the absolute value of $E_{P}$ is used.

If $E_{P}$ is larger than 1, we say demand is elastic: Consumer response is large relative to the change in price.

If $E_{P}$ is less than 1, we say demand is inelastic: Consumers are not very responsive to price changes.

If $E_{P}$ is equal to 1, demand is unitary elastic. In this case, the percentage change in quantity demanded is exactly equal to the percentage change in price.
